# On-line Ammonia Sensor and Invisible Security Ink by Fluorescent Zwitterionic Spirocyclic Meisenheimer Complex

**DOI:** 10.1038/srep40465

**Published:** 2017-01-16

**Authors:** Tanmay Das, Apurba Pramanik, Debasish Haldar

**Affiliations:** 1 Department of Chemical Sciences, Indian Institute of Science Education and Research Kolkata, Mohanpur 741246, West Bengal, India

## Abstract

Ammonia is not only a highly important gas for civilization but also contribute significantly for climate change and human health hazard. Highly sensitive ammonia sensor has been developed from a fluorescent zwitterionic spirocyclic Meisenheimer complex. Moreover, formation of this Meisenheimer complex can also be utilized for selective as well as naked eye instant detection of nitro aromatic explosive picric acid. The presence of a quaternary nitrogen atom directly attached to the spiro carbon is the unique feature of this Meisenheimer complex. This excellent photoluminescent (PL) Meisenheimer complex has two distinct stimuli responsive sites. One is sensitive towards acid while the other one is towards the base. These two positions can be modulated by adding one equivalent acid and one equivalent base to result two new products which are non fluorescent. One of these two non fluorescent species was found very exciting because of its UV/Vis transparency. Utilizing this concept we have fabricated an on-line sensor for measuring ammonia in dry or humid and condensing sewer air. The sensor was robust against ambient temperature and humidity variation. We have also developed an invisible ink from this Meisenheimer complex, with potential application for security purpose.

Ammonia (NH_3_), a low cost but efficient refrigerant is highly important gas for its application in agriculture, cold storage, fertilizer, anti-fungal agent and other chemical industries^1^,^2^,^3^. The worldwide production of ammonia is about 100 million tons. Though ammonia is highly reactive gas and vapor density is 0.59 of that of air, ammonia vapor is not flammable. The ammonia exposure (from sewer air and industrial leakage) also contribute significantly for climate change and human health hazard^4^,^5^,^6^. Exposure to 500 ppm ammonia causes immediate irritation in the moist areas of the body such as respiratory system, eyes. Higher concentration results in nasopharyngeal and tracheal burns, respiratory distress, bronchiolar and alveolar edema. There are several deadly ammonia gas disasters such as Houston’s 1976, Minot 2002. In 2015, ammonia leak mishap killed 5 and injured 140 people in Ludhiana^7^. There are also many reports of ammonia emission from cold storage and ice factory^8^. Recently, significant progress has been made in monitoring ammonia emission and sensing^9^. In comparison, little work has been done regarding the understanding of ammonia emission from sewer systems^10^. Hence, the on-line robust sensors for ammonia in dry or humid condition potentially provide a solution to the aforementioned problems.

Materials showing external stimuli controlled fluorescence output, remained in the limelight during the past few decades. The current scenario reveals the prosperous future for these fluorescent switchable molecules. These materials have already been applied in different purposes like fabrication of detector, sensing device, thermal imaging, memory and display devices^11^,^12^,^13^,^14^,^15^. Different classes of fluorescent molecules have so far been designed and synthesized in such a way that they can be used as a fluorescent switch using external stimuli like pH, light, metal ions, redox potential, temperature and vapours etc^16^,^17^,^18^,^19^,^20^,^21^,^22^,^23^. In recent decade with the increment of cyber theft, data recording and storage in a secured way has become crucial in economic as well as military fields. In this regard security ink has gained immense importance during the past few decades^24^,^25^. For application as a security ink, the components of that ink must be invisible under visible light as well as UV light. The printed material should not be photocopied and the code is readable under certain special conditions^26^. Highly luminescent materials have become of increasing importance because their emission behavior can be totally changed upon application of certain external stimuli. So these stimuli responsive photoluminescent materials are promising candidates for the application of data recording and data security^27^,^28^,^29^. Different photoluminescent materials have been used as security ink. Organic dyes, conjugated polymer dots, inorganic quantum dots are in this list. Carbon dots being easier to synthesize, highly fluorescent, with low cost and low toxicity are the latest edition in this list. But these carbon dots also have shortcomings as the code written by these materials may be visible by naked eye under ambient light^30^,^31^ or the excitation of UV light^32^,^33^. Therefore code written using materials having more confidentiality will be of much demand. In this context we have introduced a novel class of organic luminescent molecule. In the early 1960’s, a red fluorescent compound was reported from the reaction between N,N’-Dicyclohexylcarbodiimide (DCC) and picric acid with 1% yield along with 1,3-Dicyclohexyl-1-(2,4,6-trinitro-phenyl)-urea as a major product^34^,^35^,^36^. But they were unable to characterize or increase the yield of that red fluorescent compound. Initially they thought that the low yield of that red compound might have been due to the bulkiness of cyclohexyl group which was somehow preventing the transformation. So the fluorescent molecule resulting from DCC and picric acid remained as a holy grail for five long decades till now. We are first reporting this molecule with atomic level structure. The molecule is a highly fluorescent zwitterionic spirocyclic Meisenheimer complex. The guanidine nitrogen which is directly bonded to the spiro carbon holds most of the positive charge density as revealed from the crystal structure. This is the most unique feature of this Meisenheimer complex. Because in general for a zwitterionic spirocyclic Meisenheimer complex the hetero atom which forms bond with the spiro carbon should not possess any positive charge. Because this will make the molecule unstable as the sp^3^ carbon will become electron deficient and the system will be keen to rearomatize^37^. More exciting fact about this molecule was its pH dependent dual fluorescent switching. One of the nonfluorescent counterpart of our synthesized molecule was transparent under visible and UV light. So this molecule became very promising for its application as a security ink. One of the fluorescent switching pathways produces ammonia mediated colour change from colourless to fluorescent orange-red. This gave us the idea to apply this fluorescent switching mechanism in aerial ammonia sensing. Besides these applications the overall synthetic methodology was applied to detect picric acid instantly in a highly selective and sensitive manner.

## Results

**Figure 1:**
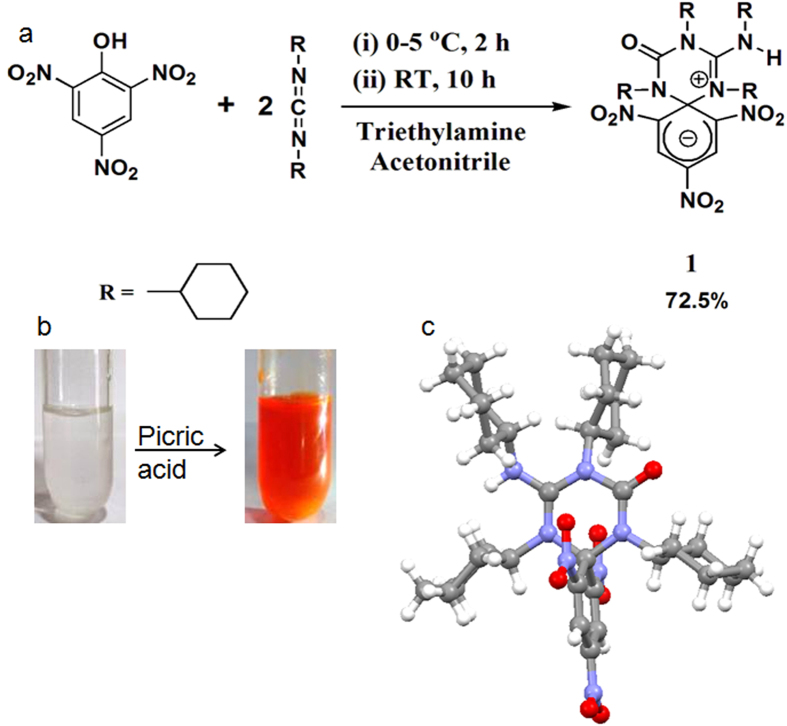
(**a**) The schematic presentation of synthesis of compound **1**. (**b**) Instant and selective detection of picric acid using DCC kit. (**c**) The solid state structure of compound **1**.

**Figure 2:**
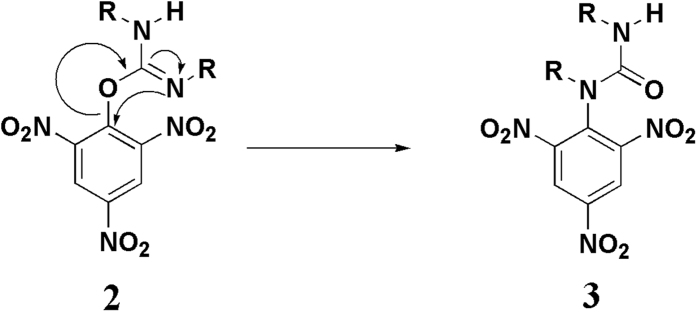
The schematic presentation of synthesis of compound 3 from 2.

We have synthesized the zwitterionic spirocyclic Meisenheimer complex **1** from DCC and picric acid (Fig. 1) and characterized using NMR, FT-IR, Mass Spectrometry and XRD. The colourless solution of DCC in acetonitrile became highly fluorescent orange-red coloured (ϕ = 0.67) instantly (Fig. 1b and Supplementary Video 1) by addition of picric acid. Even an extremely low concentration of nitro aromatic explosive picric acid can be detected using fluorescence spectroscopy. So far we have detected picric acid up to 10 ppb level. We tried to perform the same reaction between DCC and other phenols having low pk_a_ value such as o-nitrophenol, p-nitrophenol, o,p-dinitrophenol, 3,5-dinitromethylsalicylate and 3,5-dinitrosalicylaldehyde. But none of them reacted with DCC to yield a fluorescent zwitterionic spirocyclic Meisenheimer Complex like **1**. So we can conclude that this reaction is highly selective to 2,4,6-trinitrophenol system. Though **1** is a zwitterionic intermediate it is highly stable in open air as well as in presence of moisture. The reason for this exceptional stability may be the presence of four bulky cyclohexyl groups which are literally wrapping the outer surface of the molecule. The molecule **1** shows wide range of solubility in different organic solvents. We have proposed a possible mechanism (see Supplementary Figure S1) for the formation of **1** based on the well established reaction mechanism of carbodiimide mediated peptide bond formation^38^,^39^. Generally, prior to formation of peptide linkage the N-terminal protected amino acid is reacted with carbodiimide to form an O-acyl isourea at 0 °C. Then a C-terminal protected amine is added to this cold O-acyl isourea solution to result a dipeptide and N,N’-dialkyl urea. During this process there is a possible side reaction where the O-acyl isourea transforms into N-acyl urea through a rearrangement known as O-acyl isourea to N-acyl urea transformation (see Supplementary Figure S2). This side reaction can be minimized by keeping the solution at 0 °C till the amine is added. Once an N-acyl urea has been formed, no further reaction takes place. Picric acid being highly acidic its reaction with carbodiimide resembles with that of carboxylic acid and carbodiimide. In our case we have isolated 1,3-Dicyclohexyl-1-(2,4,6-trinitro-phenyl)-urea **3** which is stable and does not produce **1** upon reaction with an additional DCC molecule. Considering **2** as our first possible intermediate we have considered DCC as a nucleophile similar to the amine in case of peptide coupling to attack the ipso carbon of DCC-activated picric acid. The formation of compound **3** from the 1,3-Dicyclohexyl-2-(2,4,6-trinitro-phenyl)-isourea **2**, similar to the O-acyl to N-acyl transformation (Fig. 2) is additionally supporting our proposed mechanism^40^.

The crystal structure of compound **1** reveals that the triazine unit and 2,4,6-trinitrocyclohexadienyl anion moiety are orthogonally oriented to each other. The negative charge in the 2,4,6-trinitrocyclohexadienyl anion ring is delocalized into the conjugated NO_2_ groups (Fig. 1c). These results in the reduction of C-N (NO_2_) bond lengths compared to C-N bond lengths of picric acid. On the other side of the triazine ring we have also measured the C-N bond lengths of the substituted guanidium moiety. This reveals the delocalization of the positive charge over the three nitrogens as each C-N bond lengths are shorter than 1.47 Å which is the typical C-N single bond length. But the C-N bond length adjacent to spiro carbon is the shortest (1.33 Å) and closest to C=N double bond length. This indicates that the positive charge density is mostly residing on the N atom directly attached to the spiro carbon. From the crystal structure it also appears that there is only one molecule of **1** in the asymmetric unit and there are no other short contacts or hydrogen bond or pi-pi interaction. In higher order packing each molecule is surrounded by six molecules through hydrophobic interactions (see Supplementary Figure S3).

Solid state FT-IR spectrum of **1** shows a sharp band at 3416 cm^−1^ for N-H stretching (see Supplementary Figure S4). The position and sharpness of this peak also confirms that the N-H proton is not hydrogen bonded. Presence of extremely bulky cyclohexyl groups in **1** restricts intermolecular hydrogen bonding. Compound **1** shows two absorption maxima around 405 nm and 527 nm (see Supplementary Figure S5a). This kind of spectra is characteristic for 2,4,6-trinitrocyclohexadienyl anion system where two absorption maxima generally appear in between 400 to 600 nm depending on the substituents attached to the spiro carbon^41^. Because of the presence of three highly electron withdrawing NO_2_ groups this complex is not expected to be fluorescent. But the system has an electron rich cyclohexadienyl anion where six π-electrons are distributed over five carbons. Fluorescence studies carried on 1,1’-dihydro-2,4,6-trinitrocyclohexadienate in acetonitrile shows an emission maximum around 670 nm with a quantum yield value 0.09^42^. For compound **1** emission maxima is around 572 nm (see Supplementary Figure S5b) with a quantum yield of 0.67.

**Figure 3:**
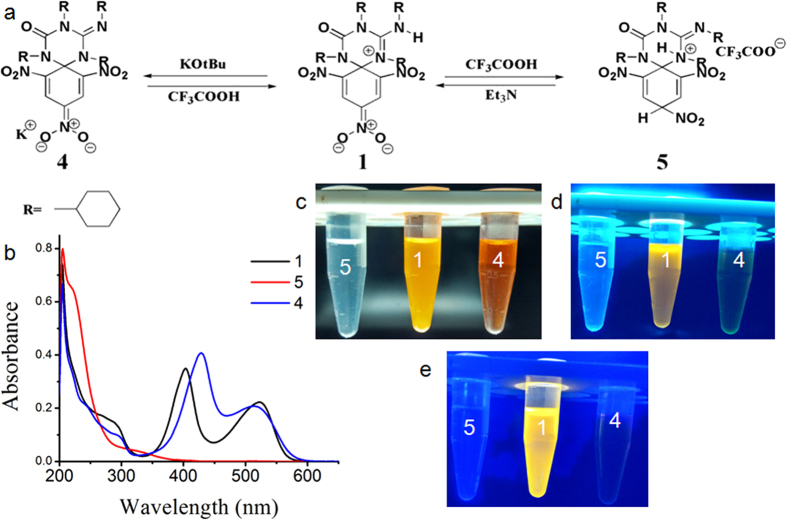
Acid and base mediated dual fluorescent switching of 1. (**a**) The schematic presentation of interconversion between **1**, **4** and **5**. (**b**) Combined absorption spectra of **1**, **4** and **5** in methanol (concentration 2.95 × 10^−5^ M). Methanolic solution of **1**, **4** and **5** under (**c**) Natural light, (**d**) 254 nm and (**e**) 366 nm irradiation.

We have shown the fluorescence switching mechanism between **1** to **4** and **1** to **5** (Fig. 3a) by studying absorption and emission spectra upon addition of potassium tert-butoxide (KOtBu) (base) and trifluoroacetic acid(TFA) (acid). Upon reaction with one equivalent of a strong base such as KOtBu, the N-H proton of guanidine moiety of triazine unit is abstracted to result compound **4**. During **1** to **4** transformation orange coloured solution of **1** becomes reddish brown coloured (Fig. 3c). **4** can regenerate **1** upon treatment of one equivalent of organic acid such as TFA (Fig. 3a). Compound **1** reacts with TFA to generate **5** and during this conversion orange coloured solution of **1** becomes transparent (Fig. 3c). The regeneration of compound **1** from **5** was achieved by adding triethylamine (Et_3_N) to the solution of **5**. These studies were performed in methanol as **1**, **4**, **5**, KOtBu, Et_3_N and TFA were soluble in methanol.

**Figure 4:**
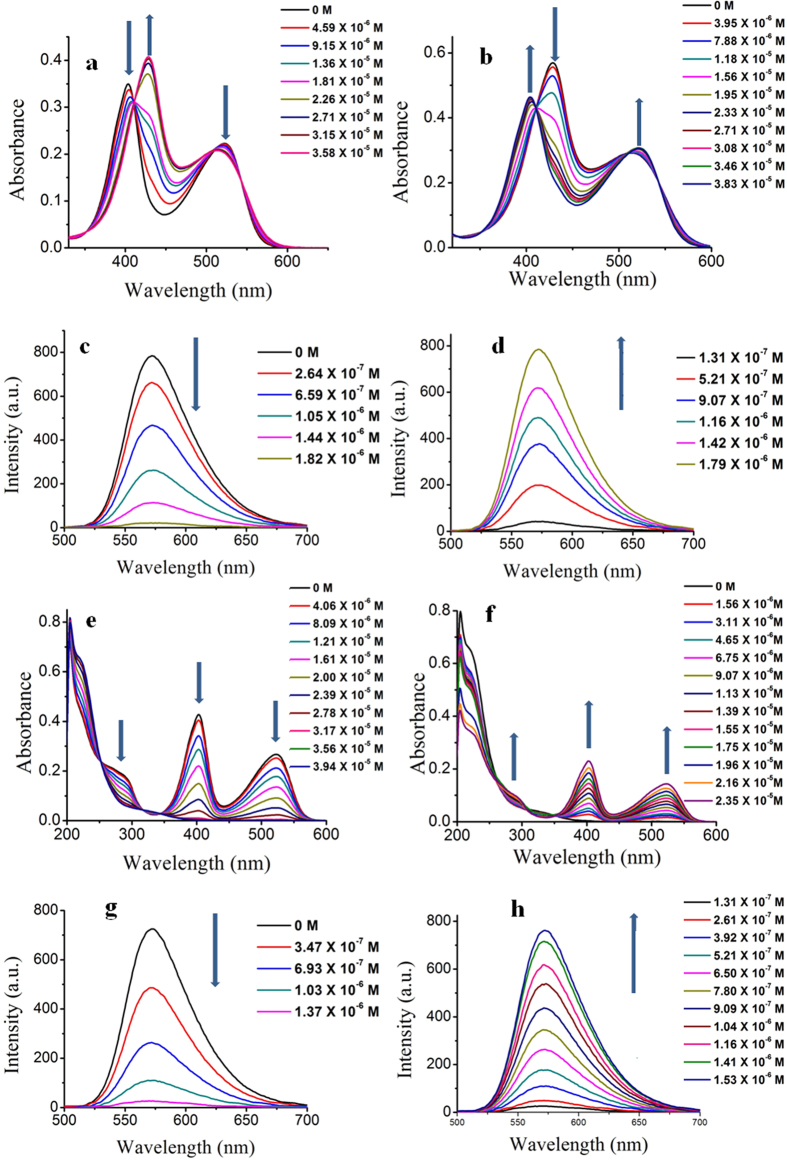
Change of absorption and emission spectra during fluorescence switching. (**a**) Change of UV/vis spectra of **1** with gradual addition of KOtBu, (**b**) Change of UV/vis spectra of **4** with gradual addition of TFA, (**c**) Fluorescence quenching of **1** with gradual addition of KOtBu, (**d**) Fluorescence turn on with gradual addition of TFA to **4**, (**e**) Change of UV/vis spectra of **1** with gradual addition of TFA. (**f**) Change of UV/vis spectra of **5** with gradual addition of Et_3_N, (**g**) Fluorescence quenching of **1** with gradual addition of TFA and (**h**) Fluorescence turn on with gradual addition of Et_3_N to **5**, (for fluorescence spectroscopy, excitation wavelength was 405 nm).

Compound **4** and **5** are non fluorescent. A solid state FT-IR spectrum of **5** shows a broad peak at 3392 cm^−1^ which is indicating the presence of an N-H proton (see Supplementary Figure S6). Figure 4a shows the change of absorption spectra upon addition of KOtBu to the methanolic solution of **1**. Titration with the base clearly shows the disappearance of **1** and generation of **4** after deprotonation of guanidine N-H of triazine moiety. Conversion of **1** to **4** was also studied by fluorescence spectroscopy (Fig. 4c). With gradual addition of base, the fluorescence intensity started to decrease due to the generation of non fluorescent compound **4**. After complete deprotonation of guanidine N-H of triazine moiety, **1** is quantitatively transformed to **4**. As a result the solution becomes non fluorescent. The regeneration of compound **1** was achieved by adding TFA to the methanolic solution of **4**. This conversion was also monitored using UV/Vis spectroscopy (Fig. 4b) and fluorescence spectroscopy (Fig. 4d). We studied this fluorescence on/off switching for ten consecutive cycles and we saw fluorescence intensity value coming back to the value almost similar to its previous cycle. Similarly we have also explored the fluorescent on/off mechanism between **1** and **5** by investigating absorption and emission spectra upon addition of trifluoroacetic acid and triethyl amine. We did these studies in methanol. Figure 4e shows the variation of absorption spectra upon addition of TFA. After certain point characteristic spectra of **1** totally vanishes and we see the spectra due to **5**. Fluorescence spectra of compound **1** were also measured with successive addition of TFA (Fig. 4g). With gradual addition of TFA the fluorescence intensity was found to diminish and at last after quantitative formation of **1** to **5** the solution becomes non fluorescent. During the generation of **5** the 2,4,6-trinitrocyclohexadienyl anion fluorophore becomes protonated and the total emission vanishes. The regeneration of compound **1** from **5** was achieved by adding Et_3_N to the solution of **5**. This conversion was also explored using UV/Vis spectroscopy (Fig. 4f) and fluorescence spectroscopy (Fig. 4h). This **1** to **5** fluorescent switching was done for twenty consecutive cycles and we observed fluorescence intensity coming back to the value almost similar to its previous cycle.

**Figure 5:**
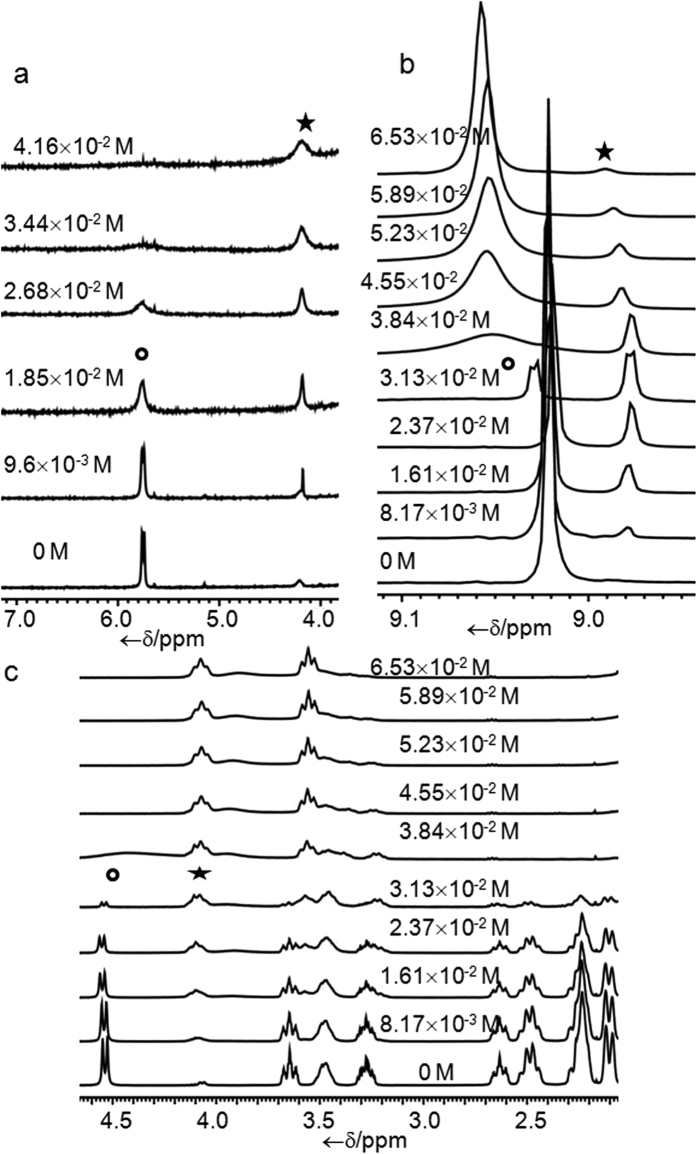
NMR titration Plots. (**a**) Part of ^1^H NMR titration of **1** with KOtBu in DMSO-*d*_*6*_, (**b**,**c**) are part of ^1^H NMR titration of **1** with TFA in CDCl_3_.

The conversion of **1** to **4** has been monitored using ^1^H NMR spectroscopy (Fig. 5a). NMR titration of **1** with KOtBu in DMSO-*d*_*6*_shows two changes. First one is the decrease of peak intensity of N-H proton at 5.75 ppm and second one is the increasing peak intensity of O-H proton due to the generation of tert-butanol at 4.15 ppm with gradually increasing concentration of KOtBu. Compound **1** is sensitive to acid also. Compound **1** reacts with one equivalent of TFA to result **5**. The conversion of **1** to **5** has also been confirmed by ^1^H NMR studies (Fig. 5b). NMR titration of **1** with TFA in CDCl_3_ reveals series of changes. First the peak at 9.1 ppm due to C-H protons of 2,4,6-trinitrocyclohexadienyl anion moiety started to split into two weakly splitted doublet (Fig. 5b). Second the peak intensity at 4.53 ppm due to N-H proton started to decrease (Fig. 5c). Third a multiplet started to appear at 4.1 ppm with increasing concentration of TFA (Fig. 5c). Overall summation of the abovementioned changes are the protonation of the para carbon of 2,4,6-trinitrocyclohexadienyl anion moiety which results the splitting of adjacent proton’s signals and deprotonation of N-H proton. Later, on further addition of TFA those weakly splitted doublets started to merge and converted to a broad singlet. While the multiplet at 4.1 ppm changes its splitting pattern and transforms into a triplet. On the other hand signal due to the N-H proton vanishes after certain point and a new broad peak at 4.15 ppm appears which is shifted towards downfield with gradual addition of TFA. This new signal is arising due to the protonation of N atom attached directly to the spiro sp^3^ carbon.

## Ammonia Sensing

**Figure 6:**
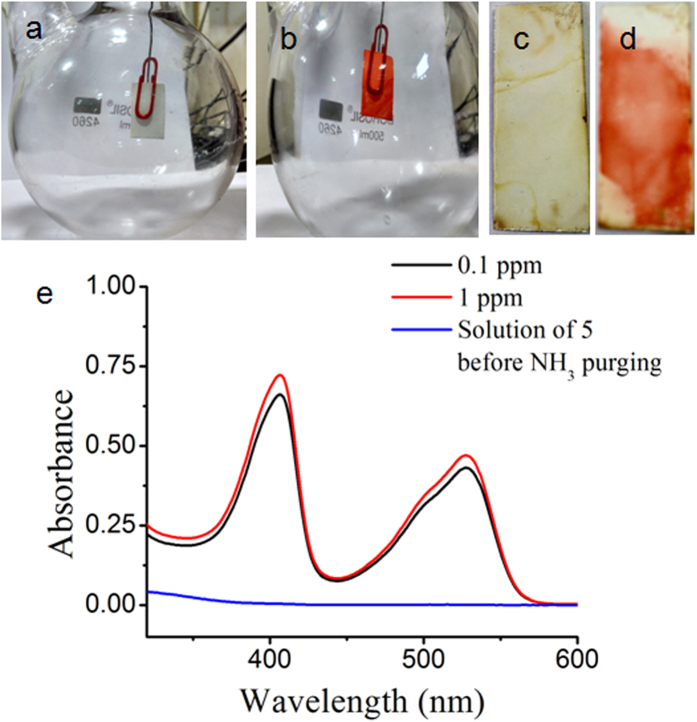
Ammonia sensing by compound 5. (**a**) Insertion of strip into hydrated ammonia chamber, (**b**) after one minute, (**c**) keeping the strip inside public urinal, (**d**) after one hour, (**e**) Absorption spectra of the solution of **5** after purging ammonia.

**Figure 7:**
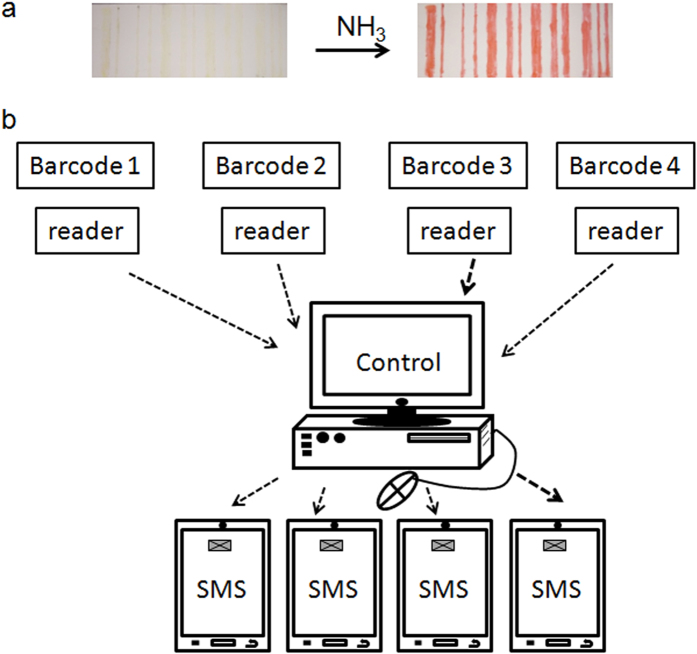
Barcode based on-line ammonia sensing. (**a**) The barcode printed on a TLC plate using compound **5** and its colour change on exposure to ammonia. (**b**) The schematic diagram of barcodes, barcode scanners (readers) and control. The on-line barcode technology can specifically detect the ammonia affected region and send a computer generated SMS.

Although ammonia is an industrially highly useful chemical, it is highly toxic^43^. The lower limit of human ammonia perception by smell is around 50 ppm. People working in ammonia environment for long time, the ammonia concentration should not be more than 20 ppm. Exposure of around 500 ppm ammonia can cause severe illness. In last few years ammonia sensing^44^,^45^,^46^,^47^,^48^,^49^ has gained immense importance due to its toxic effects on human health and climate change. Most of them are electrical sensors with detection range starting from 1 to 1000 ppm. Reports of ammonia detection below 1 ppm^50^ level are rare. But for medical application, the sensor has to be sensitive enough to detect ammonia in the ppb range. Ammonia is a potential disease biomarker^51^ and recently there are reports to identify ammonia related diseases like renal insufficiency^52^, hepatic dysfunction^53^, Helicobacter pylori infection^54^, or halitosis^55^. Moreover, ammonia sensors can be useful in environmental gas analysis, automotive industry, chemical industry and sewer treatment. So an on-line ammonia sensor will be highly appreciated. In all these applications the ammonia sensor has to detect different level of ammonia concentration. To demonstrate ammonia sensing application a strip of TLC plate was printed with the dichloromethane solution of compound **5**. The compound appeared as colourless spot on the strip. The strip was inserted into a round bottom flask containing aqueous solution of ammonia (Fig. 6a). The strip turned to orange-red immediately (Fig. 6b) (Supplementary Video 2). We have also placed the strip in a public urinal (Fig. 6c). The strip turned to orange-red after one hour (Fig. 6d). We have studied the sensitivity by spectroscopic technique. To detect ammonia quantitatively in a close room such as the public urinal, a set up has been developed. We prepared a chamber and placed a normal aquarium pump inside the chamber. Then *in situ* ammonia was generated by reacting aqueous NH_4_Cl solution with NaOH to get a desired concentration of ammonia inside the chamber. The chamber was kept undisturbed for one hour for spreading the ammonia inside the whole chamber. Then the pump was started and the air inside the chamber was passed through the transparent solution of compound **5**. Compound **5** reacts with ammonia to produce **1** which is orange-red coloured. Compound **5** does not absorb in between 400–700 nm range but compound **1** absorbs in this range with two peaks having maximum intensity at 405 and 527 nm (Fig. 6e). The formation of **1** from **5** can be monitored using UV/Vis spectroscopy. To check whether we can measure aerial ammonia quantitatively, two chambers of concentrations of ammonia 1 ppm and 0.1 ppm were prepared. A stock solution of **5** of concentration 1.1 × 10^−3^ M was prepared. Then ammonia purging was started while keeping the solution of **5** in a long test tube. Using a stopwatch the purging time for both the chambers was noted. The purging time for 0.1 ppm chamber was ten times more than that of 1 ppm chamber. Purging was continued until a visual colour change happened (Supplementary video 3). For 0.1 ppm ammonia chamber, the purging time was kept 5 minute while for 1 ppm ammonia chamber it was 30 seconds. Then absorbance value of the resulting solutions at 405 nm was noted. The absorbance values were 0.722 and 0.660 for solutions through which 1 ppm and 0.1 ppm ammonia was purged. These two values are almost close to each other. But using a sophisticated device we can minimize the error during low ppm ammonia detection. So a standard curve can be made by plotting the absorbance values versus known ammonia concentration in ppm purged for exactly same time using the above mentioned set up. For real application we have to purge the air inside a public urinal or sewer using a pump through the solution of **5**. Here the purging time should be same which was applied for the known samples while drawing the standard curve. Then we have to measure the absorbance value of that solution. Now putting the absorbance value on the standard curve we will be able to measure the unknown ammonia concentration in sewer or public toilet. We can also apply this method for medical applications. In general human breath contains ammonia less than 1 ppm. But in case of physical disorders mentioned above the ammonia level exceeds 1 ppm^56^. For this we are going to apply fluorescence spectroscopy. We are all aware of the fact that fluorescence spectroscopy is much superior than UV/Vis spectroscopy as far as extremely low concentration detection is concerned. Compound **1** which is generating from **5** is a highly fluorescent compound (ϕ = 0.67). So if patients suffering from diseases mentioned above are able to blow through a solution of **5** for a certain time then we will be able to detect the conversion of **5** to **1** by fluorescence technique. So this sensing method can be equally helpful in medical diagnosis also. The method described so far has to be done manually. But the process of ammonia detection can be upgraded to on-line process for safety related application in industries which use or produce large amount of ammonia. We have demonstrated this on-line ammonia sensing process (supplementary video 4) using barcode technology. The barcode of Arabic number 1 was printed on a TLC plate using compound **5** (Fig. 7a) which is almost colourless and unreadable under a barcode scanner. The barcode scanner is connected to a computer through Bluetooth wireless. If there is any leakage of ammonia or suddenly a rise in ammonia concentration inside a region of factory then the encrypted barcode will transform to orange red coloured (Fig. 7a) which will be detected by the barcode scanner. Instantaneously the scanned data will be sent to the security controller through wireless communication (Fig. 7b) and switch on an alarm. In mass scale, one can print barcodes using a printer and compound **5** like an ink. For bigger industries or city sewer system spread in different sectors, one can track the environment of every individual section using this online barcode technology based ammonia sensing (Fig. 7b). In comparison with the existing ammonia sensors, some points are important. First of all, moisture often causes interference to electrical sensors but in our case there is no effect of moisture. Secondly, our highly fluorescent sensor is able to detect ammonia in the ppb range which makes it highly promising. Third one is the low cost of this sensor because of low cost precursors and one step synthetic pathway. But the most important part is this online ammonia sensing using barcode technology which can specifically detect the ammonia affected region and send a computer generated SMS with all details to each individual working in that industry. So, it is not necessary to ring an alarm and make people panicked. Rather handle the situation smoothly by evacuating the ammonia affected region only.

## Invisible Security Ink

**Figure 8:**
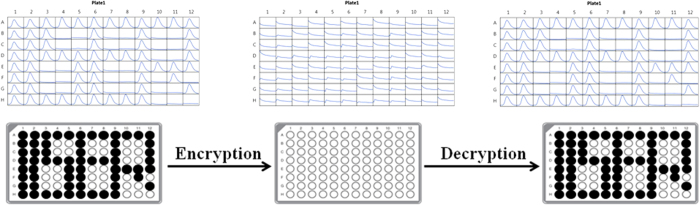
Fluorescence reading after coding, encryption and decryption using a fluorescent microplate reader.

In this paper we have already presented fluorescent on/off mechanism between compounds **1** and **5**. Compound **5** is transparent to visible light, 254 nm UV light or 366 nm UV light (see Supplementary Figure S7). So a code written using a solution of **5** will not be visible by naked eye under ambient light or 254 nm or 366 nm. It can’t be detected using fluorescence spectroscopy. So a code written by this material is totally confidential. When we need to decode the message we have to blow ammonia gas over the script. Commercially available drawing paper was used to demonstrate the application of compound **5** as a security ink (see Supplementary Figure S8). We have used a solution of **5** of concentration 10^−5^ M. After decryption the generated spots of compound **1** is not visible by naked eye as we have used a very dilute solution. But being fluorescent it can be detected using fluorescent microplate reader. This property makes our compound to be a promising candidate as a security purpose invisible ink. At first a fluorescent microplate with 96 well was taken and a trace paper was placed over the microplate and 96 circles were drawn right above their position carefully. Now the commercially available drawing paper was cut exactly the size of that fluorescent microplate. Then the trace paper was placed above the well cut drawing paper and using a HB graphite pencil the circles were redrawn over their position. After drawing 96 circles the trace paper was removed. It was clearly seen that the drawing paper was having 96 well impacted circles. Then a stock solution of concentration 6.54 × 10^−5^ M of compound **1** was prepared in dichloromethane and 5 μL of that solution was dropped over few circles to create a certain pattern. It was the coding step. At that point the drawing paper was inserted inside the fluorescent microplate reader and its fluorescence signal was recorded. Then the drawing paper was hanged using a clip inside a chamber having trifluoroacetic acid at the bottom for 30 minutes. It was the process of encryption. After that the paper was again inserted inside the fluorescent microplate reader to record its fluorescence signal. Next the paper was hanged similarly inside a chamber filled with 25% ammonia at the bottom for 5 minute. That was the decryption procedure. Then the fluorescence of that paper was measured for the last time using fluorescent microplate reader. Here we have written IISER on that drawing paper. The recorded fluorescence reading after coding, encryption and decryption is shown in Figure 8. Conversion between **1** and **5** being reversible we can encrypt or decrypt the message for several numbers of cycles.

## Discussion

Different organic poly nitro aromatics are in the list of hazardous^57^,^58^ as well as strong explosive. There are lots of reports of detecting this poly nitro aromatics^59^,^60^ but selective detection is much less. Among the highly explosive poly nitro aromatic compounds picric acid is the most devastating one. Although there are reports of selective detection of picric acid^61^,^62^,^63^,^64^,^65^,^66^, most of them are not sensitive enough to detect very low level (ppb level) of picric acid. Here we have reported the selective detection of picric acid. The colourless DCC solution in acetonitrile became highly fluorescent orange-red coloured (ϕ = 0.67) instantly by contact with picric acid up to 10 ppb level. But the other phenols having low pk_a_ value such as o-nitrophenol, p-nitrophenol, o,p-dinitrophenol, 3,5-dinitromethylsalicylate and 3,5-dinitrosalicylaldehyde have failed to form such fluorescent zwitterionic spirocyclic Meisenheimer Complex like **1**. Therefore we have proposed a kit containing 1 g DCC dissolved in 10 ml acetonitrile and 0.5 ml triethylamine for *in situ* selective picric acid detection using this current reaction. So far we have reported a new kind of highly fluorescent compound **1** which may be further employed as a biomarker after proper functionalisation. We have also explored the dual fluorescence on/off mechanism of compound **1** by application of acid and base separately. Based on this fluorescence switching we have successfully applied this material as security purpose invisible ink which can be used for data security purposes. The aerial ammonia sensing mechanism can be utilized in fertilizer plants for ammonia detection in a quantitative way. As we started our synthesis with picric acid and the resulting compound turned out highly fluorescent, we applied this methodology for detection of picric acid. With the help of fluorescence spectroscopy we were able to detect picric acid in the ppb level. The detection method turned out to be very fast (only few seconds) for visual detection in the ppm level.

In conclusion, this study revealed that a fluorescent zwitterionic spirocyclic Meisenheimer complex is suitable for sensing ammonia in dry or humid and condensing sewer air. The sensor was robust against ambient temperature and humidity variation. The NH_3_ gas response and recovery mechanism of the sensor was also explained in detail. This photoluminescent Meisenheimer complex is sensitive towards both acid and base and results two new products which are non fluorescent. Using this concept, we have also developed an invisible security ink from this Meisenheimer complex. Moreover, formation of this Meisenheimer complex itself can be utilized for selective as well as naked eye instant detection of nitro aromatic explosive picric acid.

## Methods

### Materials

Picric acid and DCC (N,N’-Dicyclohexylcarbodiimide) were purchased from Sigma Aldrich. HPLC grade acetonitrile and triethylamine were purchased from Merck and SRL chemicals respectively. Picric acid was obtained in its moist form. So this was air dried for one week prior to use. All these chemicals were used directly without further purification. The progress of the reaction was monitored by TLC. Merck TLC plates (TLC Silica gel 60 F_254_) were used for thin layer chromatography purpose. 60–120 mesh silica gel was used for column chromatography purpose. The mobile phase for the column chromatography was ethyl acetate-hexane mixture.

### NMR experiments

All NMR studies were carried out on Bruker AVANCE 500 MHz and JEOL 400 MHz spectrometer at 278 K. Compound concentrations were in the range 1–10 mM. Before recording the spectra the required sample was taken in an eppendorf and dissolved in 0.5 mL of either CDCl_3_ or DMSO-*d*_*6*_ and then transferred to the NMR tube to record the spectra.

### FT-IR spectroscopy

All reported FT-IR spectra were obtained with a Perkin Elmer Spectrum RX1 spectrophotometer. Before recording the spectra the sample (less than 1 mg) was mixed well with solid KBr. The powdered solid was then converted into a pellet using a hydraulic press. Then the pellet was placed on the holder inside the spectrophotometer to record the FT-IR spectra.

### Mass Spectrometry

Sample of desired interest was dissolved in HPLC grade methanol to prepare a μM range solution. Q-Tof Micro YA263 high-resolution (Waters Corporation) mass spectrometer was used for recording the mass spectra. Positive-mode electrospray ionization method was applied for the experiment.

### UV/Vis spectroscopy

UV/Vis absorption spectra were recorded on a Perkin Elmer UV/Vis spectrophotometer (Lambda 35). During this absorption spectra measurement 1 cm path length quartz cell was used.

### Fluorescence spectroscopy

All the fluorescence spectra including fluorescence quenching and regeneration were recorded on a Perkin Elmer fluorescent spectrometer (LS 55). 1 cm path length quartz cell was used during the measurement.

### X-ray crystallography

Good quality orange-red crystals of Zwitterionic Spirocyclic Meisenheimer complex **1** was obtained from their respective ethyl acetate-dichloromethane solution by slow evaporation. Crystallographic data of compound **1**: C_32_H_47_N_7_O_7_, Mw = 641.77, monoclinic, space group P21/n, a = 10.9266(6), b = 26.6855(13), c = 11.9495(5) Å, α = 90°, β = 11.858(6)°, γ = 90°, V = 3233.8(3) Å^3^, Z = 4, dm = 1.318 Mgm^−3^, T = 293 K, R1 0.0552 and wR2 0.1348 for 7126 data with I > 2σ(I). Intensity data were collected with MoKα radiation using Bruker APEX-2 CCD diffractometer. Data were processed using the Bruker SAINT package and the structure solution and refinement procedures were performed using SHELX97. The non-hydrogen atoms were refined with anisotropic thermal parameters. The data for compound **1** has been deposited at the Cambridge Crystallographic Data Centre with reference number CCDC 1481699.

### Fluorescent microplate reader

SpectraMax M2e fluorescent microplate reader was used to read the fluorescence signal for security ink based application.

## Additional Information

**How to cite this article**: Das, T. *et al*. On-line Ammonia Sensor and Invisible Security Ink by Fluorescent Zwitterionic Spirocyclic Meisenheimer Complex. *Sci. Rep.* **7**, 40465; doi: 10.1038/srep40465 (2017).

**Publisher's note:** Springer Nature remains neutral with regard to jurisdictional claims in published maps and institutional affiliations.

## Supplementary Material

Supplementary Video 1

Supplementary Video 2

Supplementary Video 3

Supplementary Video 4

Supporting Information
