# Supplementary material for: On-line Ammonia Sensor and Invisible Security Ink by Fluorescent Zwitterionic Spirocyclic Meisenheimer Complex
Source: Sci Rep. 2017 Jan 16;7:40465. doi: 10.1038/srep40465 (PMC5238453; doi:10.1038/srep40465)
Supplement: Supporting Information [file srep40465-s5.doc]

**Supporting information**

**On-line Ammonia Sensor and Invisible Security Ink by Fluorescent Zwitterionic Spirocyclic Meisenheimer Complex**

Tanmay Das, Apurba Pramanik and Debasish Haldar*

Department of Chemical Sciences, Indian Institute of Science Education and Research Kolkata, Mohanpur 741246, West Bengal, India

Corresponding author: Debasish Haldar, E-mail: [deba_h76@iiserkol.ac.in](mailto:deba_h76@iiserkol.ac.in), Tel: +919748487503.

**Table of contents**

| ESI Figure S1 | 2 |
| --- | --- |
| ESI Figure S2 | 2 |
| ESI Figure S3 | 3 |
| ESI Figure S4 | 3 |
| ESI Figure S5 | 4 |
| ESI Figure S6 | 4 |
| ESI Figure S7 | 5 |
| ESI Figure S8 | 5 |
| Figure S1 | 6 |
| Figure S2 | 6 |
| Figure S3 | 7 |
| Figure S4 | 7 |
| Figure S5 | 8 |
| Figure S6 | 8 |
| Figure S7 | 9 |
| Figure S8 | 9 |
| Experimental | 10 |
| Photophysical Measurements | 10-11 |
| Legends for video 1, 2, 3 and 4 | 11 |


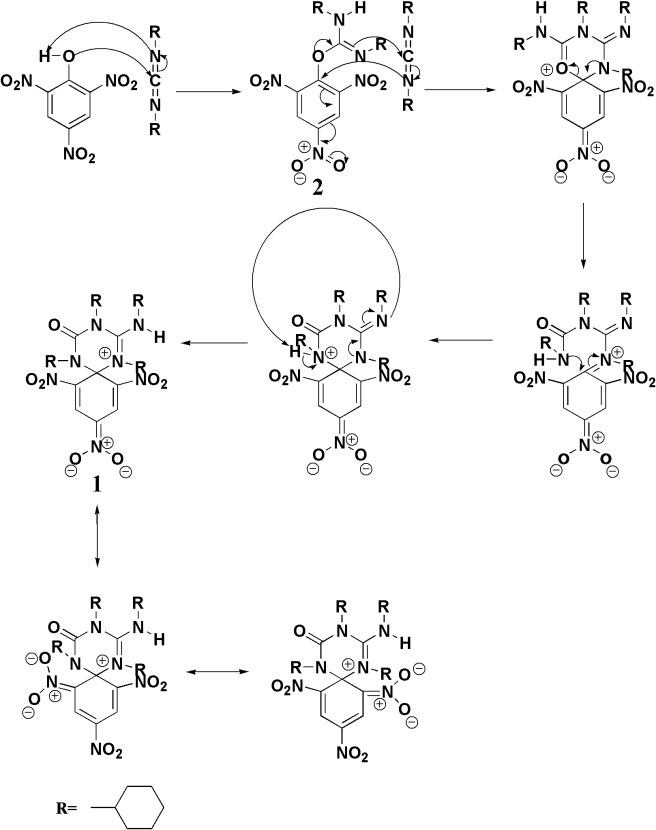


**ESI Figure S1:** Possible mechanism for the formation of compound **1.**

**
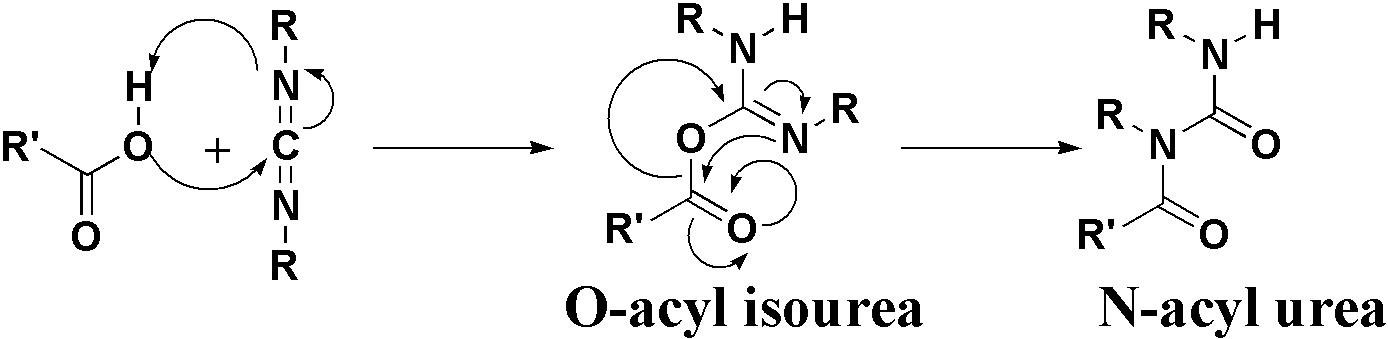
**

**ESI Figure S2:** O-acyl isourea to N-acyl urea transformation.


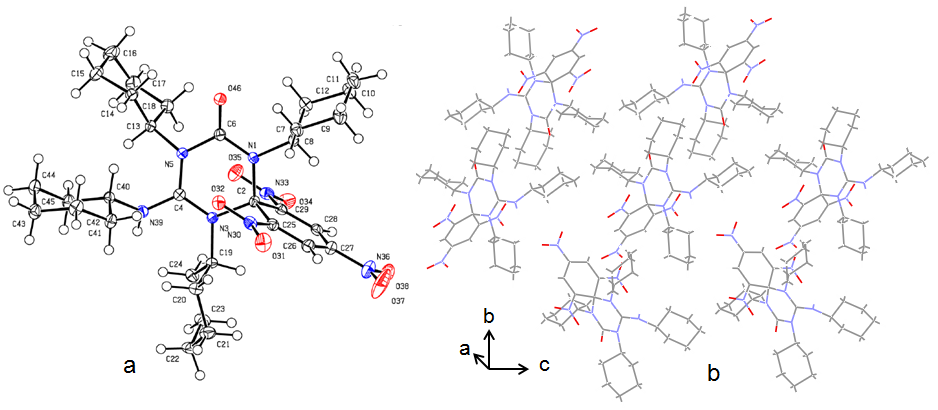


**ESI Figure S3:** a) ORTEP diagram of **1**. b) Higher order packing of compound **1** where each molecule is surrounded by six other molecules.


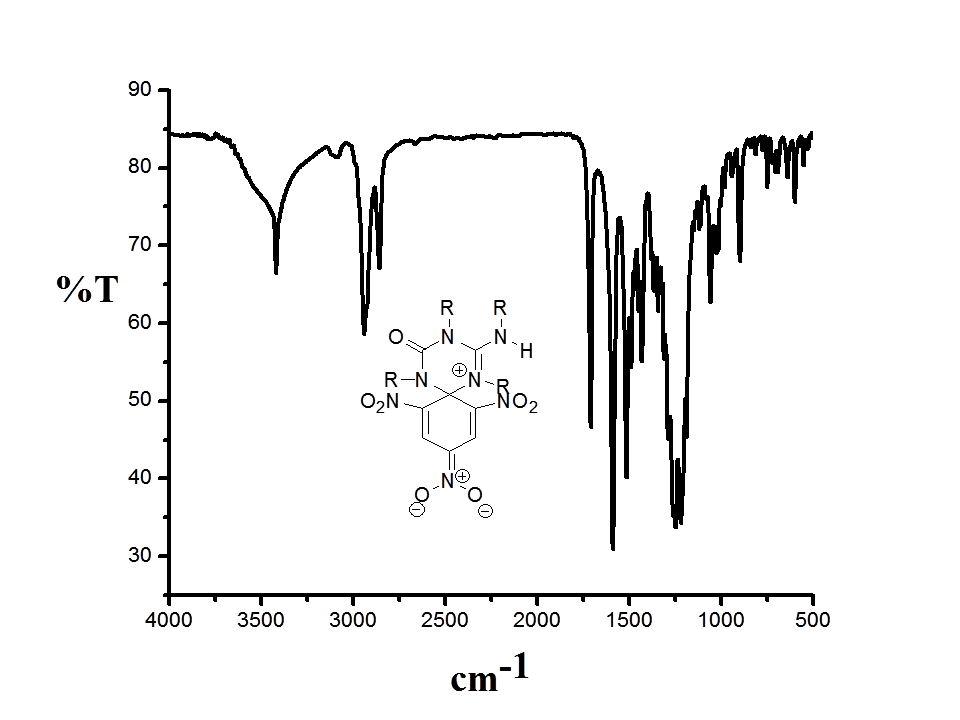


**ESI Figure S4:** IR spectra of ZSM complex **1**.


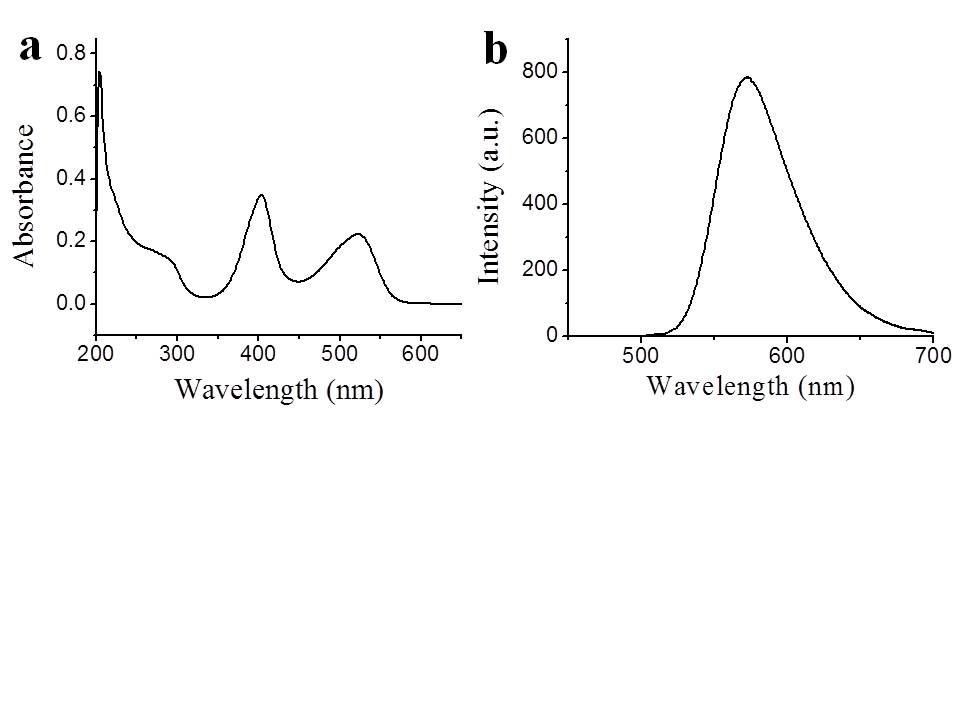


**ESI Figure S5:** (a) Absorption spectra (2.95 X 10^-5^ M in dichloromethane) and b) Emission spectra (5.9 X 10^-6^ M in dichloromethane) (λ_ex_. = 405 nm).


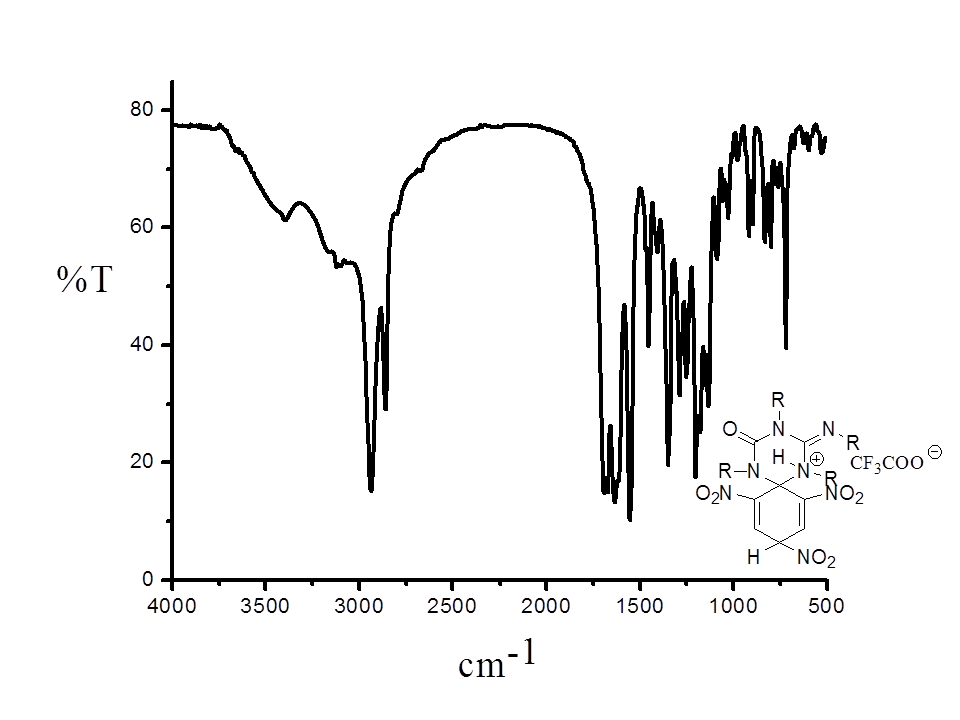


**ESI Figure S6:** IR spectra of **5**.


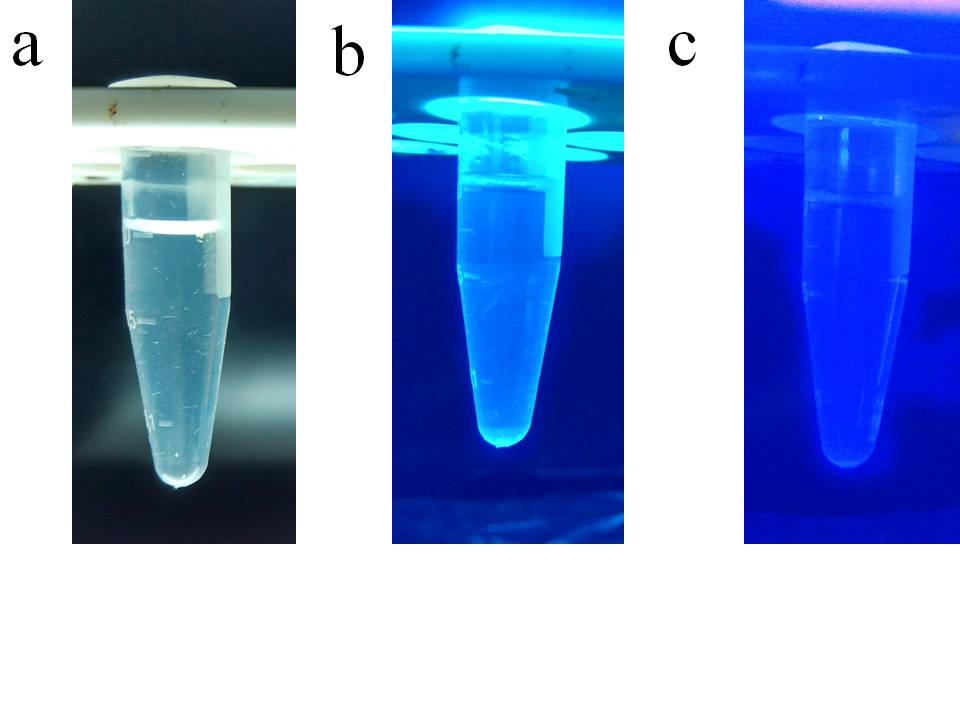


**ESI Figure S7:** Solution of compound **5** under (a) Natural light, (b) 254 nm and (c) 366 nm irradiation.


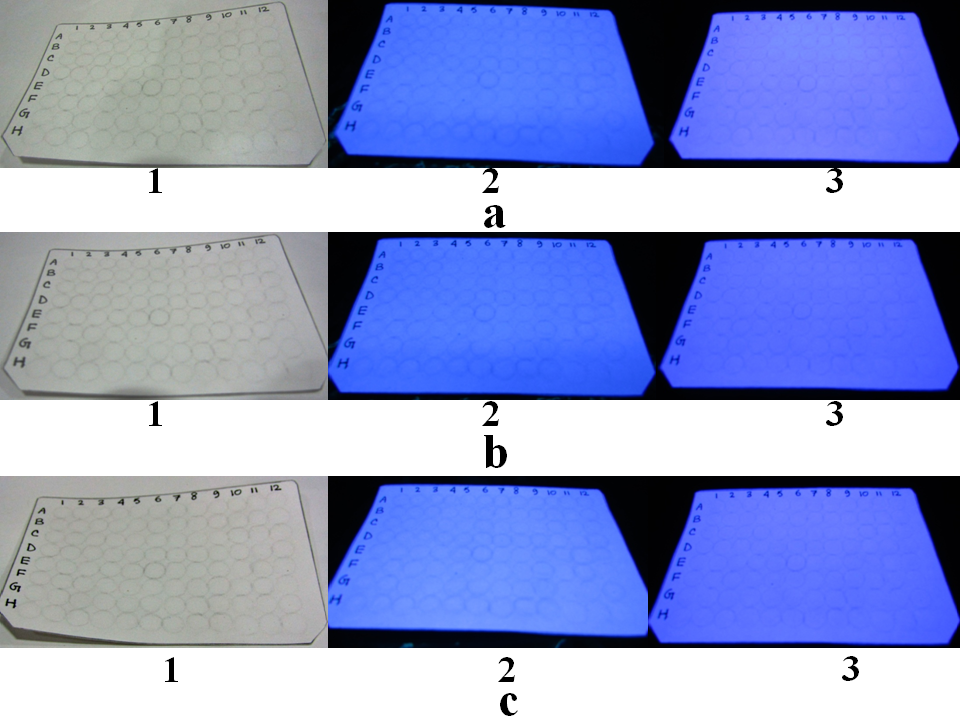


**ESI Figure S8:** Image of the 96 circled paper of the size of fluorescent microplate. **a**, **b** and **c** refers coding, after encryption and after decryption respectively. While **1**, **2**, and **3** refers under natural light, 254 nm UV light and 366 nm UV light.


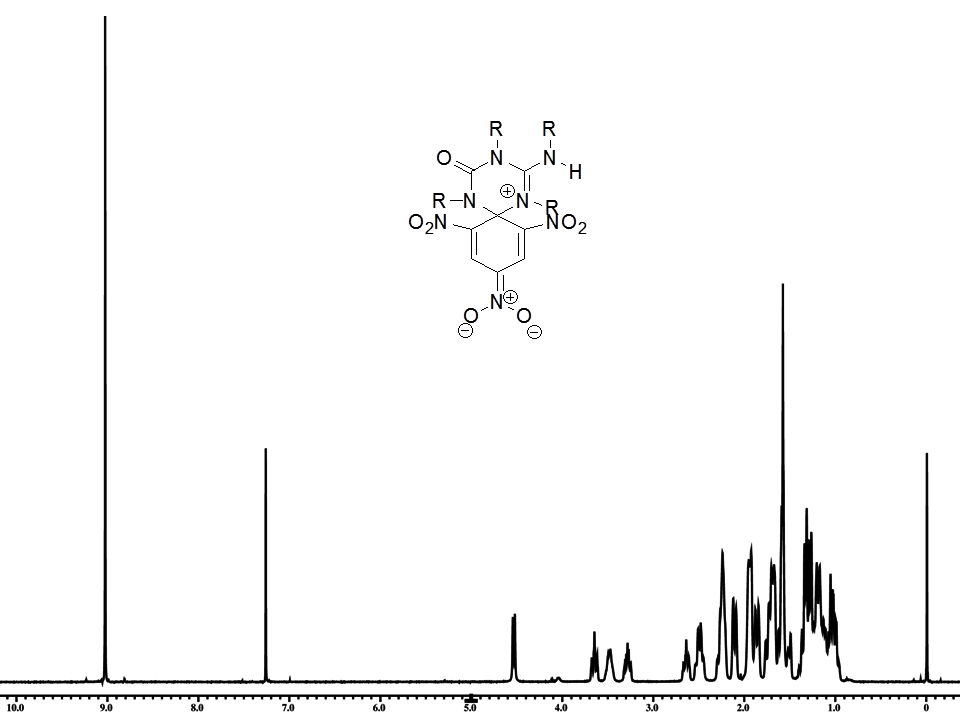


**Figure S1:** ^1^H NMR (CDCl_3_, 400 MHz, *δ* in ppm) spectra of ZSM complex **1**.


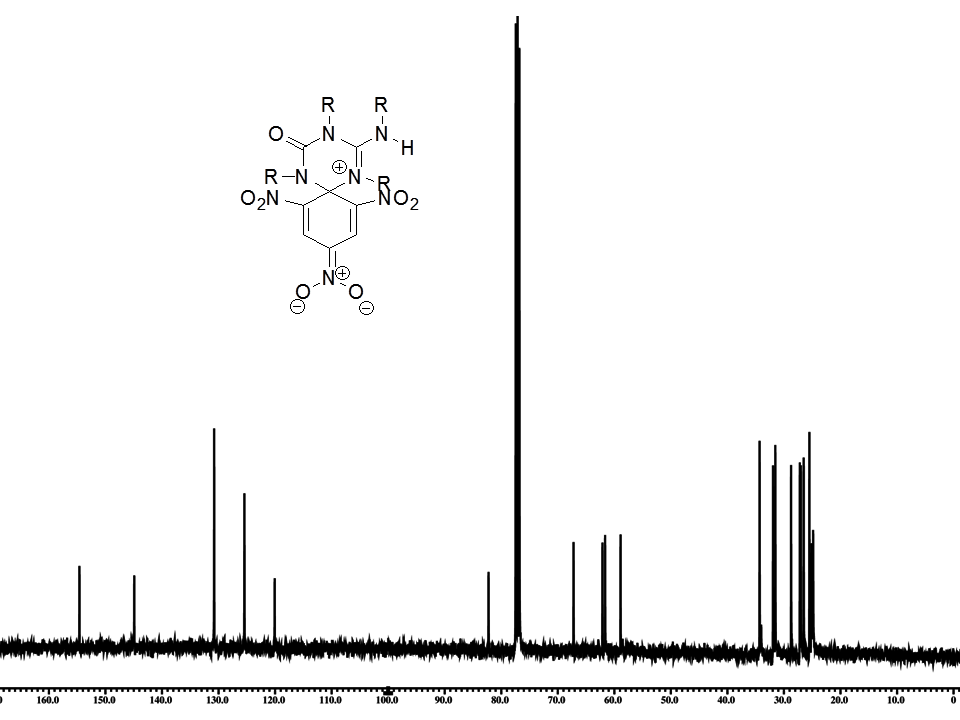


**Figure S2:** ^13^C NMR (CDCl_3_, 100 MHz, *δ* in ppm) spectra of ZSM complex **1**.


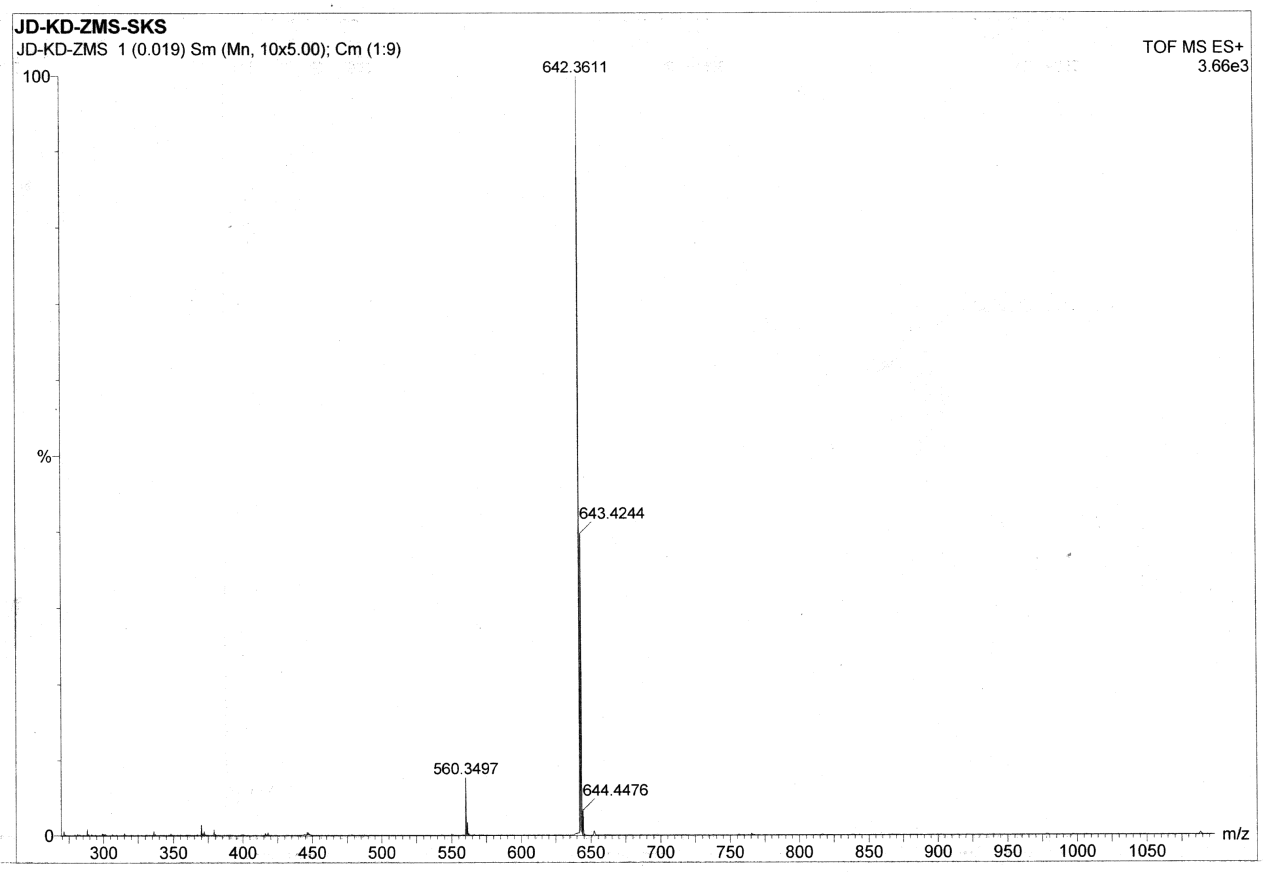


**Figure S3:** Mass spectra of **1**.


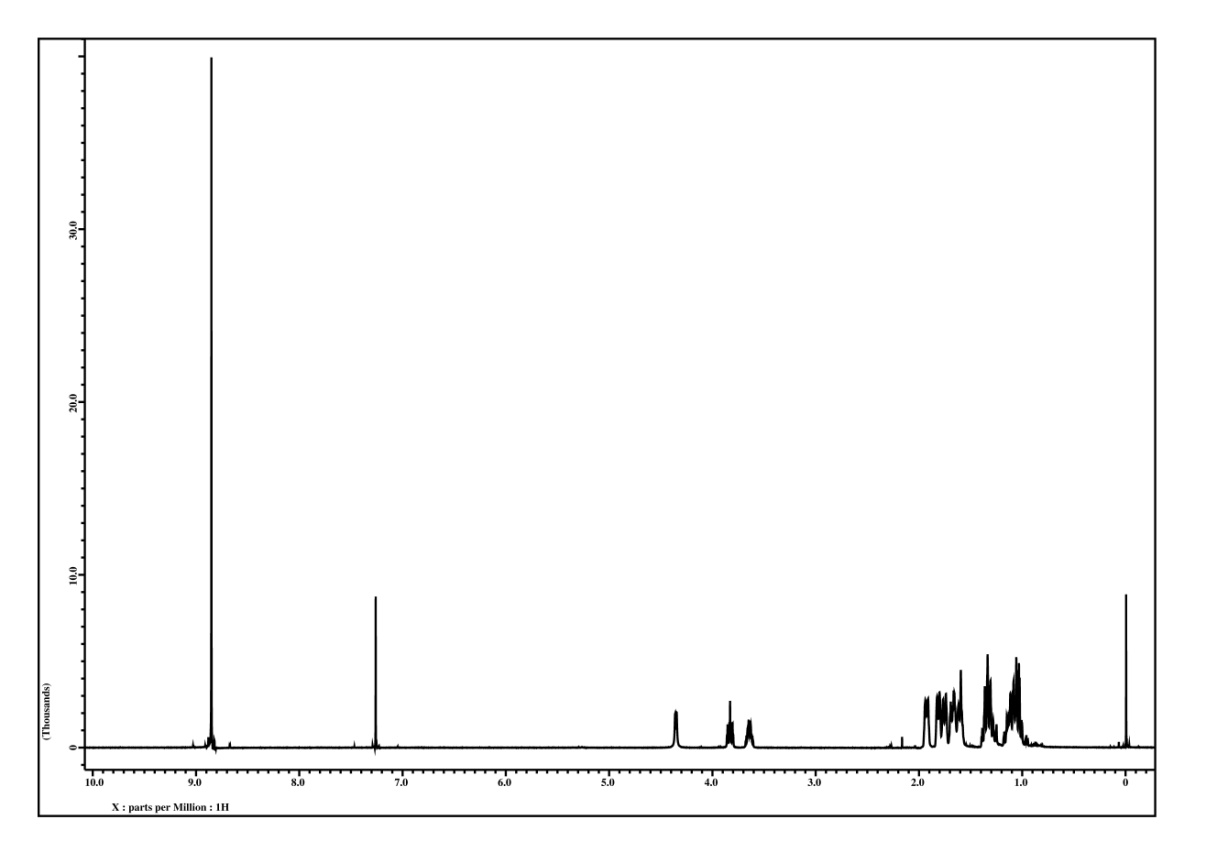


**Figure S4:** ^1^H NMR (CDCl_3_, 500 MHz, *δ* in ppm) spectra of **3**.


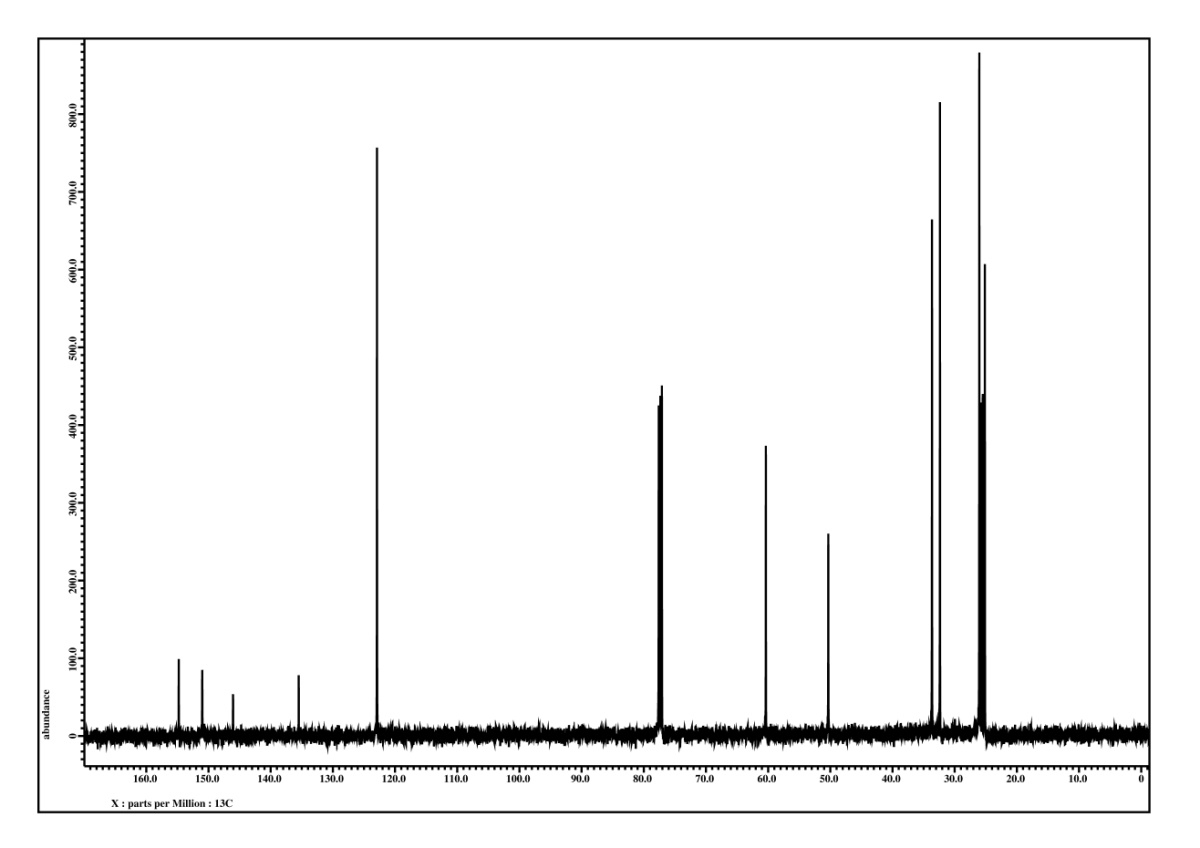


**Figure S5:** ^13^C NMR (CDCl_3_, 125 MHz, *δ* in ppm) spectra of **3**.


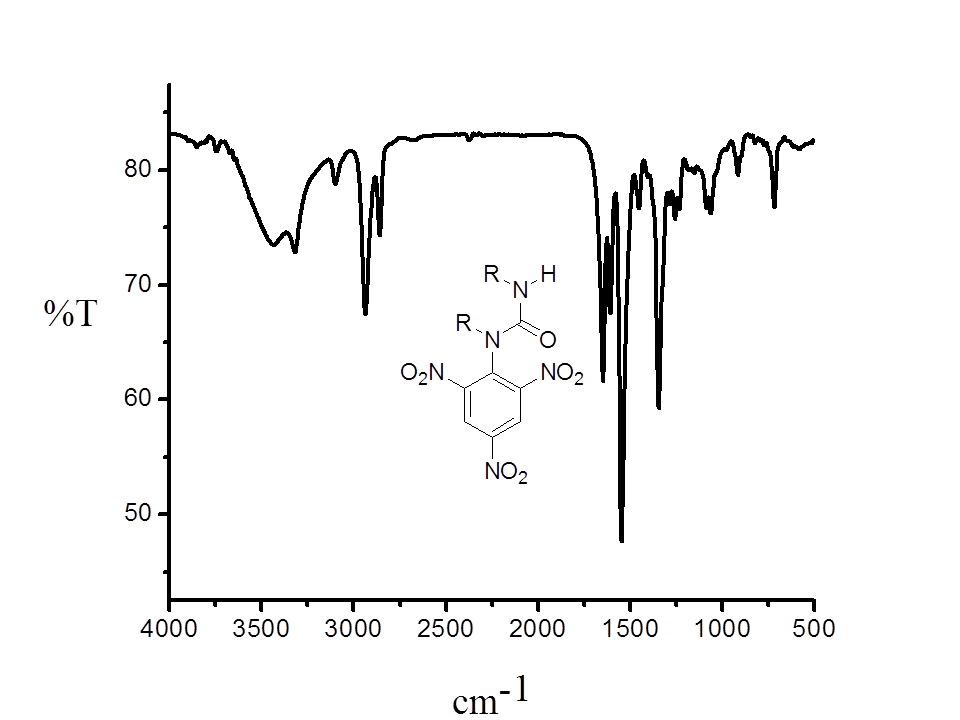


**Figure S6:** IR spectra of **3**.


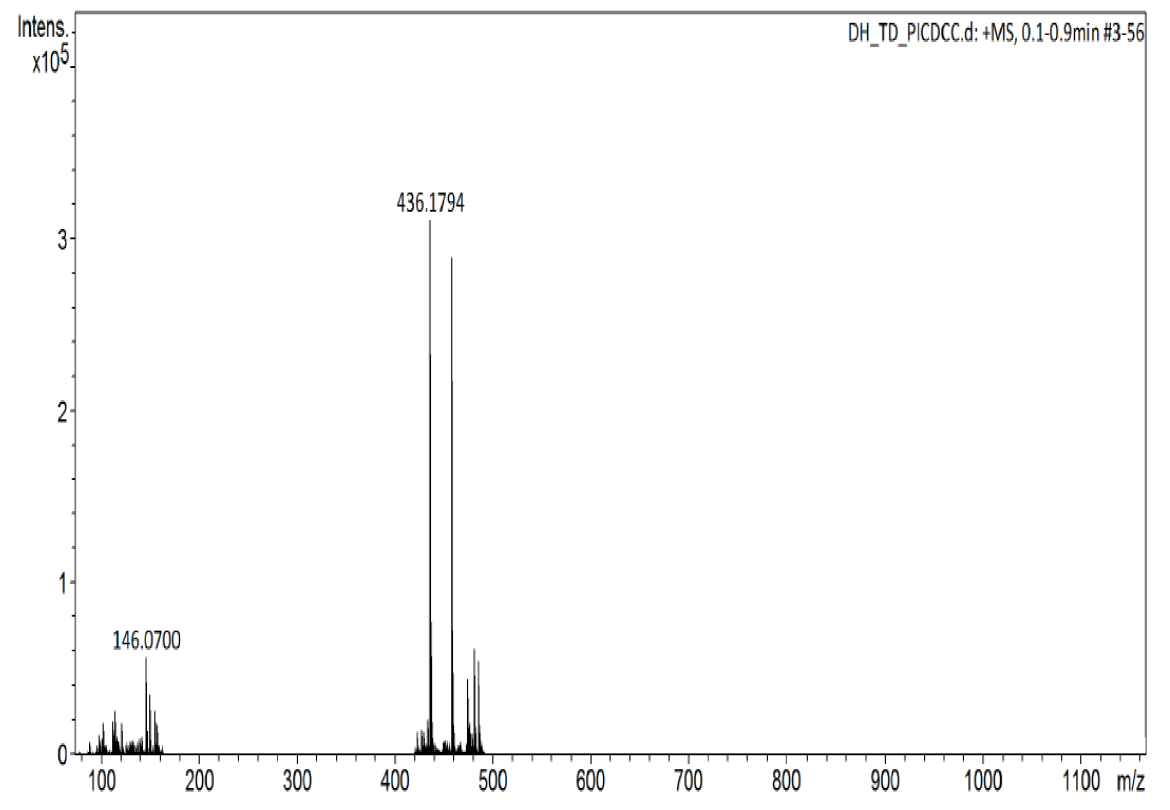


**Figure S7:** Mass spectra of **3**.

**
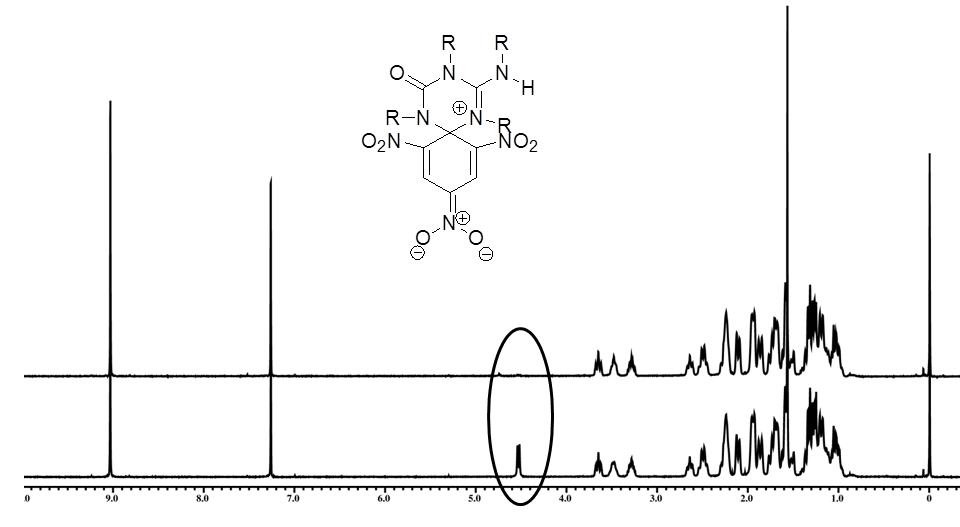
**

**Figure S8:** Plot of ^1^H NMR (CDCl_3_, 400 MHz, *δ* in ppm) spectra of ZSM complex **1** and after deuterium exchange using D_2_O to confirm N-H proton.

**Experimental**

**Synthesis of Compounds 1 and 3:**

Picric acid (3 g, 13.1 mmol) was dissolved in HPLC grade acetonitrile (150 mL) and cooled in an ice water bath such that the temperature remains in between 0-5^o^ C. After cooling the solution for 20 minutes N,N'-dicyclohexylcarbodiimide (13.5 g, 65.5 mmol, 5.0 equiv.) and triethylamine (2 mL, 14.4 mmol) were added to it all at a time. Then the mixture was stirred for two hours for 0-5^0^ C. After two hours the solution was allowed to come at room temperature and stirred for additional ten hours. During this time period sufficient amount of orange solid separated from the solution which was filtered and washed with acetonitrile to get pure product. The filtrate was removed under reduced pressure and the crude product was dissolved in 250 mL dichloromethane. The orange luminescent solution of dichloromethane was extracted with dilute sodium carbonate solution to remove unreacted picric acid. Then the dichloromethane layer was collected, dried over anhydrous sodium sulphate and evaporated under reduced pressure. The orange crude product was purified by column chromatography using silica gel (60-120 mesh) and ethyl acetate/n hexane (1:2) as eluent. First eluting compound was **3** and this was collected as a yellow solid after evaporation of solvent. Second eluting compound **1** was collected as orange red solid. **1** was crystallized from dichloromethane and ethyl acetate mixture.

Yield: 6.1 g (9.51 mmol, 72.5 % for compound **1**) and 0.28 g (0.655 mmol, 5 % for compound **3**).

For compound **1**: ^1^H NMR (400 MHz, CDCl_3_, δ ppm): 9.02 (2H, s), 4.53 (1H, d, 9.2 Hz), 3.69-3.59 (1H, tt), 3.54-3.42 (1H, m), 2.69-2.58 (1H, tt), 2.56-2.41 (2H, q, J = 10 Hz), 2.32-2.17 (4H, m), 2.15-2.06 (2H, d, J = 12 Hz), 2.0-1.9 (4H, m), 1.9-1.81 (2H, d, J = 14.6 Hz), 1.79-1.61 (6H, m), 1.61-1.55 (4H, t), 1.54-1.47 (2H, m), 1.4-1.23 (6H, m), 1.23-1.09 (4H, m), 1.09-0.94 (4H, m).

^13^C NMR (100 MHz, CDCl_3_, δ ppm): 154.82, 145.13, 130.99, 125.65, 120.28, 82.47, 67.43, 62.52, 61.85, 59.12, 34.53, 32.15, 31.75, 28.97, 27.41, 27.23, 26.73, 25.73, 25.42, 25.11, 25.06.

FTIR (cm^-1^): 3416, 1705, 1580, 1505, 1423, 1244, 1218, 1053, 888.

HRMS (m/z): [M]^+^ calcd. for C_32_H_47_N_7_O_7_, 641.3537; found, 642.3611 [M+H]^+^.

For compound **3**: ^1^H NMR (500 MHz, CDCl_3_, δ ppm): 8.85 (2H, s), 4.35 (1H, d, J = 6 Hz), 3.87-3.78 (1H, tt), 3.69-3.59 (1H, m), 1.98-1.88 (2H, dd), 1.81 (2H, d, J = 9.2 Hz), 1.75 (2H, d, J = 11.2 Hz), 1.72-1.54 (4H, m), 1.4-1.23 (4H, m), 1.19-0.98 (6H, m).

^13^C NMR (125 MHz, CDCl_3_, δ ppm): 154.78, 151.01, 146.05, 135.49, 122.9, 60.37, 50.32, 33.65, 32.37, 26.03, 25.83, 25.49, 25.15. FTIR (cm^-1^): 3320, 1649, 1546, 1451, 1341, 1252, 1053, 916, 717. HRMS (m/z): [M]^+^ calcd. for C_19_H_25_N_5_O_7_, 435.1754; found, 436.1794 [M+H]^+^.

**Photophysical Measurements**

The fluorescence quantum yield of compound **1** was measured taking rhodamine 6G as standard. A set of five samples for both rhodamine 6G (in ethanol) and compound **1** (in dichloromethane) were prepared having absorbance value less than 0.05. Then their absoption and emission spectra were measured. The excitation wavelength was 527 nm for both the compounds. The fluorescence quantum yield of compound **1** was calculated according to the following equation:


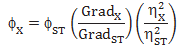


Here the subscripts X and ST stands for the test sample and the standard sample. Grad is the gradient obtained by plotting integrated fluorescence intensity vs absorbance and η is the refractive index of the solvent in which the measurements were performed.

**Supplementary video 1. Naked eye detection of picric acid.** Addition of picric acid to the transparent solution of N,N’-Dicyclohexylcarbodiimide and triethylamine in acetonitrile makes it orange red immediately.

**Supplementary video 2. Detection of aerial ammonia.** Insertion of a strip coated with compound 5 inside an ammonia chamber. Conversion of **5** to **1** causes the appearance of orange colour on that strip.

**Supplementary video 3. Detection of very low quantity aerial ammonia.** Purging of ammonia filled air from a closed chamber through the solution of **5**. Conversion of **5** to **1** causes the appearance of orange colour of the solution which was transparent initially.

**Supplementary video 4. On-line detection of ammonia using barcode technology.** In presence of ammonia barcode written using compound 5 converted to orange coloured. A barcode scanner collects the signal and transfers it to the control through Bluetooth wireless.
